# Supplementary material for: Deletion of wfs1 Impairs Oligodendrocyte Precursor Cells Dorsal Distribution and Myelination Through the wfs1-hmgcs1 Axis in Zebrafish
Source: Biology (Basel). 2026 Aug 16;15(16):1402. doi: 10.3390/biology15161402 (PMC13510151; doi:10.3390/biology15161402)
Supplement: Supplementary file 1 [file biology-15-01402-s001.zip › File S1-Transcriptome sequencing data and differentially expressed genes (DEGs) in wfs1-- zebrafish at 5 dpf/1.GO/wfs1KOvsWT/GSEA/wfs1KO/MANNOSIDASE_ACTIVITY/MANNOSIDASE_ACTIVITY(GO_0015923).html]

Details for gene set MANNOSIDASE\_ACTIVITY(GO:0015923)[GSEA]


|  || Dataset | deg.sample |
| Phenotype | sample.cls |
| Upregulated in class | wfs1KO |
| GeneSet | MANNOSIDASE\_ACTIVITY(GO:0015923) |
| Enrichment Score (ES) | 0.67459595 |
| Normalized Enrichment Score (NES) | 2.5194356 |
| Nominal p-value | 0.0 |
| FDR q-value | 0.011000001 |
| FWER p-Value | 0.0 |
Table: GSEA Results Summary

  

Fig 1: Enrichment plot: MANNOSIDASE\_ACTIVITY(GO:0015923)
  
Profile of the Running ES Score & Positions of GeneSet Members on the Rank Ordered List

  

| PROBE | DESCRIPTION   (from dataset) | GENE SYMBOL | GENE\_TITLE | RANK IN GENE LIST | RANK METRIC SCORE | RUNNING ES | CORE ENRICHMENT || 1 | 566132 | ces2 |  |  | 19 | 4.953 | 0.2038 | Yes |
| 2 | 560651 | ces3 |  |  | 137 | 4.571 | 0.3887 | Yes |
| 3 | 565117 | cel.2 |  |  | 426 | 3.195 | 0.5115 | Yes |
| 4 | 450083 | gla |  |  | 814 | 2.368 | 0.5971 | Yes |
| 5 | 322481 | cel.1 |  |  | 1043 | 2.053 | 0.6746 | Yes |
| 6 | 561967 | si:ch211-93f2.1 |  |  | 3511 | 0.928 | 0.6354 | No |
| 7 | 445105 | lipia |  |  | 7175 | 0.475 | 0.5401 | No |
| 8 | 566078 | man1a1 |  |  | 7623 | 0.445 | 0.5444 | No |
| 9 | 566040 | notum2 |  |  | 9963 | 0.305 | 0.4836 | No |
| 10 | 541519 | man2b1 |  |  | 10713 | 0.260 | 0.4708 | No |
| 11 | 394164 | edem1 |  |  | 11342 | 0.225 | 0.4604 | No |
| 12 | 792904 | si:ch73-139e5.2 |  |  | 11574 | 0.212 | 0.4619 | No |
| 13 | 335538 | man1a2 |  |  | 11788 | 0.202 | 0.4635 | No |
| 14 | 560219 | man2a1 |  |  | 12647 | 0.157 | 0.4431 | No |
| 15 | 114549 | ache |  |  | 13778 | 0.103 | 0.4118 | No |
| 16 | 100333987 | man2a2 |  |  | 14195 | 0.083 | 0.4022 | No |
| 17 | 569595 | man2c1 |  |  | 16388 | -0.014 | 0.3340 | No |
| 18 | 393128 | manba |  |  | 16754 | -0.024 | 0.3235 | No |
| 19 | 100009622 | zgc:158619 |  |  | 16788 | -0.026 | 0.3235 | No |
| 20 | 559811 | edem3 |  |  | 16984 | -0.033 | 0.3188 | No |
| 21 | 562939 | si:ch211-282j22.3 |  |  | 17674 | -0.061 | 0.2997 | No |
| 22 | 335124 | ppme1 |  |  | 18725 | -0.110 | 0.2713 | No |
| 23 | 402801 | edem2 |  |  | 18763 | -0.112 | 0.2747 | No |
| 24 | 100148657 | si:ch211-71n6.4 |  |  | 18898 | -0.118 | 0.2754 | No |
| 25 | 447827 | manea |  |  | 20041 | -0.168 | 0.2464 | No |
| 26 | 566679 | si:ch211-30b16.2 |  |  | 20711 | -0.199 | 0.2337 | No |
| 27 | 569736 | plbd1 |  |  | 23042 | -0.330 | 0.1742 | No |
| 28 | 560778 | naga |  |  | 24171 | -0.404 | 0.1554 | No |
| 29 | 562592 | man1b1a |  |  | 25693 | -0.502 | 0.1284 | No |
| 30 | 100003570 | man2b2 |  |  | 27071 | -0.637 | 0.1114 | No |
| 31 | 334017 | pla1a |  |  | 29170 | -0.964 | 0.0854 | No |
Table: GSEA details
[plain text format]

  

Fig 2: MANNOSIDASE\_ACTIVITY(GO:0015923)
  
Blue-Pink O' Gram in the Space of the Analyzed GeneSet

  

Fig 3: MANNOSIDASE\_ACTIVITY(GO:0015923): Random ES distribution
  
Gene set null distribution of ES for
 **MANNOSIDASE\_ACTIVITY(GO:0015923)**
